# Ocular Causes of Abnormal Head Position: Strabismus Clinic Data

**DOI:** 10.4274/tjo.42068

**Published:** 2017-08-15

**Authors:** Kadriye Erkan Turan, Hande Taylan Şekeroğlu, İrem Koç, Esra Vural, Jale Karakaya, Emin Cumhur Şener, Ali Şefik Sanaç

**Affiliations:** 1 Hacettepe University Faculty of Medicine, Department of Ophthalmology, Ankara, Turkey; 2 Ortaköy State Hospital, Ophthalmology Clinic, Aksaray, Turkey; 3 Mardin State Hospital, Ophthalmology Clinic, Mardin, Turkey; 4 Hacettepe University Faculty of Medicine, Department of Biostatistics, Ankara, Turkey; 5 Private Practice, Ankara, Turkey

**Keywords:** Abnormal head position, nystagmus, Ocular, Strabismus

## Abstract

**Objectives:**

To determine the most common ocular causes and types of abnormal head position (AHP) and describe their clinical features.

**Materials and Methods:**

Patients with AHP who had been followed in the strabismus unit were retrospectively reviewed. Demographic features and orthoptic characteristics were recorded.

**Results:**

A total of 163 patients including 61 women (37.4%) and 102 men (62.6%), with a mean age of 19.9±18.3 were recruited. The most common causes of AHP were determined as fourth cranial nerve palsy (33.7%), Duane retraction syndrome (21.5%), sixth cranial nerve palsy (11%), nystagmus blockage syndrome (9.8%) and Brown syndrome (6.7%). Other less frequent causes were A-V pattern strabismus, comitant strabismus, thyroid orbitopathy and third cranial nerve palsy. The most common types of AHP were head tilt (45.4%) and face turn (36.8%). Out of 142 patients whose visual acuity could be evaluated, 28.2% had amblyopia. The frequency of amblyopia varied depending on the diagnosis (p<0.001), while there was no relation between amblyopia and different types of AHP (p=0.497). Stereopsis and fusion could be tested in 128 patients and 43.8% of them had stereopsis and fusion. The presence of stereopsis and fusion was found to be related with the diagnosis (p=0.001), whereas it was not related with the types of AHP (p=0.580). The presence of amblyopia was not significantly associated with fusion (p=1.000) or stereopsis (p=0.602).

**Conclusion:**

There are many ocular pathologies that cause AHP. Patients with similar diagnoses may have different types of AHP. Patients may have amblyopia and impaired binocularity despite AHP. Therefore, all patients with AHP should be examined in detail and these points should be considered in the treatment plan.

## INTRODUCTION

Abnormal head position (AHP) refers to the head forming an angle with the body on horizontal, vertical or anteroposterior axis.^1^ AHP may occur due to ocular, muscular, neurological, or vestibular causes.^2^ When examining a patient with AHP symptoms in the clinic, the cause of the position can be distinguished from orthopedic and vestibular causes by simply having the patient close their eyes and observing the correction of the position.^2^ Alterations in normal head alignment may manifest as the chin looking upwards or downwards, the face being turned to the right or left, the head being tilted right or left, or various combinations of these positions.^1^ AHP of ocular origin includes head malpositions resulting from false information obtained from afferent vision paths, oculomotor nerves, or the vestibular aparatus.^1^ Although the underlying causes of AHP vary, ocular AHP is a mechanism developed in order to increase visual acuity, optimize visual field, ensure single and binocular vision or fusion, and prevent diplopia.^3,4^ Persistent AHP due to ocular pathology may lead to permanent deformities caused by muscular atrophy and musculoskeletal system changes secondary to the position.^5^

This study aimed to evaluate the AHP types and etiologies in AHP patients being followed in the strabismus unit, and to determine the relationship between AHP and clinical findings.

## MATERIALS AND METHODS

This retrospective study was conducted in accordance with the principles of the 2013 Declaration of Helsinki and with the consent of the Hacettepe University Non-interventional Clinical Research Ethics Board. The medical records of patients being followed in the strabismus unit were reviewed. Patients who had a history of ocular surgery or whose AHP was of non-ocular origins were not included in the study. A total of 163 patients met these eligibility criteria and were included in the analysis. The patients’ age, gender, AHP type, AHP degree (°), best corrected visual acuity, amount of deviation (prism diopters [PD]), ocular motility findings, and binocularity were recorded. Visual acuity was measured using Snellen or Lea chart and expressed in the decimal system. Strabismus measurements were made using the Krimsky test or prism cover test. In patients who complied with examination, fusion was assessed with the Worth 4 dot test and stereopsis with the Titmus stereo test. Fusion and stereopsis were assessed without AHP correction. AHP was measured on three axes using orthopedic goniometry.

### Statistical Analysis

Descriptive statistics were expressed in mean ± standard deviation for continuous numerical variables and in number and percentage for categorical variables. Correlations between categorical variables were assessed using chi-square test (Fisher’s exact or Yates corrected chi-square). Statistical analyses were performed using IBM SPSS statistics for Windows, version 21.0 (IBM Corp., Armonk, NY, USA) software. P values less than 0.05 were considered statistically significant.

## RESULTS

Of the 163 patients, 61 (37.4%) were female and 102 (62.6%) were male. The mean age was 19.9±18.3 years (1-73 years). The most common diagnoses in patients with AHP were fourth nerve palsy (33.7%), Duane retraction syndrome (21.5%), sixth nerve palsy (11%), nystagmus blockage syndrome (9.8%), and Brown syndrome (6.7%). The frequency distribution of the diagnoses and the ophthalmologic examination findings are summarized in Table 1. Among all patients, the AHP types in order of prevalence were head tilt (45.4%), face turn (36.8%), combined AHP (11.7%), chin up (5.5%) and chin down (0.6%). Each diagnostic group showed different AHP types with one being predominant. The mean degree of head tilt was 18.92±7.08° (10-45°) and the mean degree of face turn was 20.30±9.04° (5-40°). The mean degree of chin up position was 19.22±7.45° (8-35°), whereas the one patient with chin down position showed 10° tilt.

Of the 142 patients with measurable visual acuity, 40 (28.2%) had amblyopia. The average visual acuity was 0.83±0.22 (0.1-1.0). Amblyopia was most common in nystagmus blockage syndrome (100%) and least common in Duane retraction syndrome (16.0%). There was a significant correlation between diagnosis and the incidence of amblyopia (p<0.001). However, there was no significant difference in amblyopia prevalence among the various AHP types (p=0.497).

In primary gaze position, 14.7% of the patients were orthotropic. The most common type of strabismus was esotropia (28.2%). The mean amount of deviation was 24.18±15.81 PD (3-60 PD) in the 52 esotropic patients and 23.39±15.41 PD (3-65 PD) in the 43 exotropic patients. The mean amount of deviation was 14.86±7.74 PD (4-40 PD) in the 74 patients with vertical strabismus. Forty-five patients had diplopia. One hundred and thirty-nine patients (85.3%) exhibited varying degrees of ocular motility limitation. Twelve of those patients had clinically insignificant bilateral/symmetric minimal movement restriction, despite the absence of any paralytic or restrictive etiology.

Of the 128 patients who could be assessed for fusion and stereopsis, 43.8% had both fusion and stereopsis. There was no statistical difference in fusion or stereopsis rates among the various AHP types (p=0.580), but there was a significant difference according to diagnosis (p=0.001). Analysis of fusion and stereopsis in the diagnostic groups revealed significantly high rates of stereopsis and fusion loss (93.3%) in the sixth nerve palsy group (p=0.001). When fusion and stereopsis were considered separately, no significant difference was found among the various AHP types (p=0.352 for fusion, p=0.702 for stereopsis), but a significant difference emerged between diagnoses (p<0.001 for fusion, p=0.013 for stereopsis). Amblyopia was not significantly associated with the presence of fusion (p=1.000) or stereopsis (p=0.067). There was no significant correlation between the degree of AHP and fusion (p=0.378), stereopsis (p=0.611), or amblyopia (p=0.065).

## DISCUSSION

The clinical detection of AHP due to ocular causes is very important for several reasons, including the possibility of developing secondary and permanent torticollis as a result of muscular and soft tissue changes due to delayed treatment, loss of binocularity that may occur if the AHP cannot be maintained, and development of amblyopia.^3,6^ AHP is among the important diagnostic criteria for paralytic diplopia and nystagmus.^7^ Various series evaluating the causes of AHP have listed the most common ocular causes. Mitchell^8^ reported incomitant strabismus in 52.4%, nystagmus in 19%, and congenital esotropia in 10.9% of 630 patients with ocular torticollis. In the same study, the most common causes of incomitance were identified as A-V pattern, fourth nerve palsy, asymmetric surgery, Duane retraction syndrome, and Brown syndrome.^8^ Incomitant strabismus was also a prominent cause of ocular AHP in the present study, and the five most common causes were fourth nerve palsy (33.7%), Duane retraction syndrome (21.5%), sixth nerve palsy (11%), nystagmus blockage syndrome (9.8%), and Brown syndrome (6.7%). Dikici and Kızılkaya^9^ found that AHP was a result of some type of strabismus in 80% of 187 patients, and 80% of all the cases were incomitant. In another study of 64 patients with Down syndrome and AHP, incomitant strabismus was reported as the most common identifiable cause (26.6%).^10^ In their review of 2,701 participants who presented to an ophthalmology clinic due to any ophthalmologic complaint, Erkan Turan et al.^11^ determined that 30 patients had AHP and emphasized that comitant strabismus, nystagmus, and Duane retraction syndrome were the most common causes of AHP.

The presence and the type of AHP is important in diagnosing ocular disease.^12^ Boricean and Bărar^13^ determined face turn to be the most common type of AHP in a study of children. However, in our study, head tilt was the most common (45.4%), followed by face turn (36.8%). The diagnostic distribution of the patients included in a study is the most important factor influencing the frequency of AHP. Head tilt was the most common type of AHP in the present study because the most common diagnosis in our patient group was fourth nerve palsy. In their study presenting the clinical characteristics and surgical treatment of 75 patients with Duane retraction syndrome, Kalevar et al.^14^ reported that 86% of esotropic patients and 80% of the exotropic patients had AHP in primary position, while there was no AHP in the orthotropic patient group. Biler Demirkılınç et al.^15^ reviewed patients with Duane retraction syndrome and emphasized that the most common clinical finding was AHP. Suh et al.^16^ reported that 12 of 13 patients with Brown syndrome had AHP and classified the cases as slight, medium, or severe depending on the amount of AHP and the presence of vertical strabismus in the gaze positions. In their study presenting the surgical outcomes of patients with unilateral superior oblique palsy, Tenlik et al.^17^ detected AHP in 97.3% of 37 patients. In our study, we observed that different types of AHP can occur in patients with the same diagnosis (Table 1). Therefore, all diagnoses should be considered and investigated in patients presenting with AHP.

As AHP is a compensating mechanism, it is believed that fusion capacity and visual acuity that stimulates fusion are both necessary. Consequently, patients with amblyopia or suppression may not be expected to develop AHP.^18^ Similarly, a heterotropia that cannot be balanced with position or fusion amplitudes can be considered a factor that causes amblyopia.^6^ While we did not find a significant correlation between the presence of amblyopia and binocularity and the type of AHP, we found that the prevalence of amblyopia and binocularity vary depending on diagnosis. Stereopsis is a high-order binocular function.^19^ Stereopsis may be absent despite motor and sensory fusion, and stereopsis may be present, though rarely, without motor fusion.^19^ We did not evaluate motor fusion in the present study because we had a very wide age range in our patient group. However, since stereopsis and fusion are cortical functions at different levels, we assessed their mutual interaction with AHP individually and found that AHP was not significantly correlated with either of them. Although not common, AHP may accompany comitant strabismus. In cases of infantile esotropia, amblyopia, and severe fixation preference, head position can be improved, especially in reading and focused gaze positions.^7^ In pattern strabismus, a position that minimizes strabismus and provides binocularity may be preferred.^20^ AHP can also be observed in non-strabismus cases such as uncorrected refractive error.^21^ It should be kept in mind that the group of patients in the study did not have a homogenous diagnosis distribution and that binocular function could not be assessed in all patients. Furthermore, we measured fusion using the Worth 4 dot test and stereopsis with the Titmus test. Using different tests for these measurements may yield different results. Therefore, these findings may not be sufficient to explain the origin of AHP in all patients sharing the same diagnosis, and cannot be generalized to all strabismus patients. In order to determine whether the presence of AHP is protective with respect to amblyopia and loss of binocularity and to explain AHP pathogenesis on an individual basis, studies should be conducted on AHP and non-AHP patients with the same diagnosis. Patients without AHP were not included in our study.

### Study Limitations

Our study has the limitations of any retrospective work. In this study, we evaluated patients with AHP who were being followed in the strabismus unit. Therefore, it is not possible to generalize the results to include all patients having AHP. Because it was not possible to evaluate binocularity and visual acuity in all of the patients included in the study, these analyses do not encompass all patients. In order to individually examine why patients with different diagnoses use AHP, studies that include larger patient groups and obtain detailed and reliable fusion and stereopsis measurements from patients with and without AHP are needed.

## CONCLUSION

Different types of AHP may occur in patients with the same diagnosis. Patients with AHP should be examined for different diagnoses. It should be kept in mind that predictions regarding amblyopia and the presence of binocularity cannot be made based on AHP type, and that the patient’s diagnosis should also be considered during evaluation. Another thing to remember is that the presence of amblyopia may not always be accompanied by loss of binocularity.

## Figures and Tables

**Table 1:**
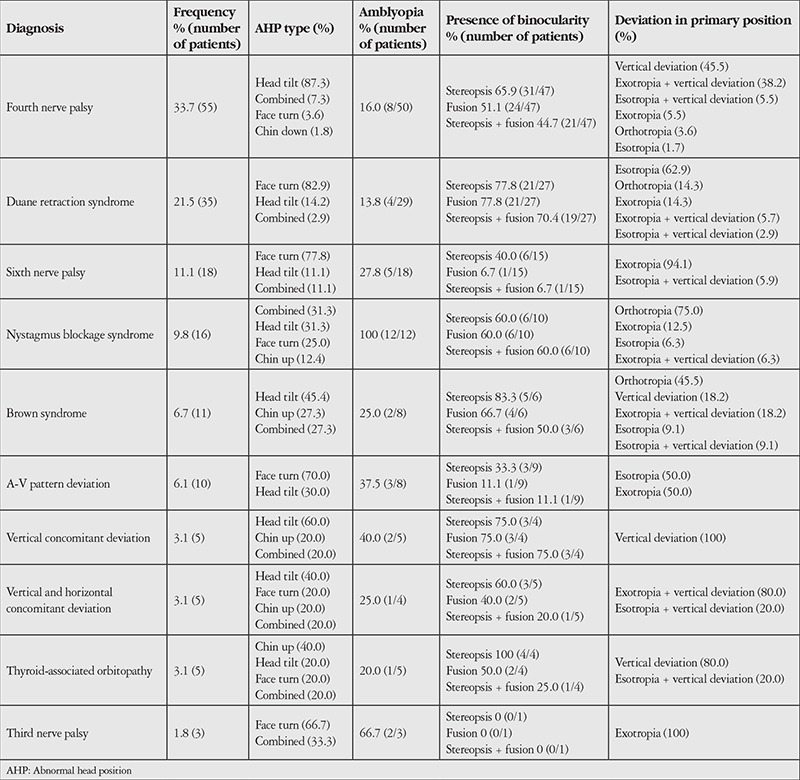
The frequency of abnormal head positions and examination findings according to diagnosis (n=163)
